# Performance Development in Adolescent Track and Field Athletes According to Age, Sex and Sport Discipline

**DOI:** 10.1371/journal.pone.0129014

**Published:** 2015-06-04

**Authors:** Espen Tønnessen, Ida Siobhan Svendsen, Inge Christoffer Olsen, Atle Guttormsen, Thomas Haugen

**Affiliations:** 1 Norwegian Olympic Federation, Oslo, Norway; 2 School of Sport, Exercise and Health Sciences, Loughborough University, Loughborough, United Kingdom; 3 Diakonhjemmet, Oslo, Norway; 4 University of Life Sciences, Ås, Norway; Victoria University, AUSTRALIA

## Abstract

**Introduction:**

Sex-specific differences that arise during puberty have a pronounced effect on the training process. However, the consequences this should have for goal-setting, planning and implementation of training for boys and girls of different ages remains poorly understood. The aim of this study was to quantify performance developments in athletic running and jumping disciplines in the age range 11-18 and identify progression differences as a function of age, discipline and sex.

**Methods:**

The 100 all-time best Norwegian male and female 60-m, 800-m, long jump and high jump athletes in each age category from 11 to 18 years were analysed using mixed models with random intercept according to athlete.

**Results:**

Male and female athletes perform almost equally in running and jumping events up to the age of 12. Beyond this age, males outperform females. Relative annual performance development in females gradually decreases throughout the analyzed age period. In males, annual relative performance development accelerates up to the age of 13 (for running events) or 14 (for jumping events) and then gradually declines when approaching 18 years of age. The relative improvement from age 11 to 18 was twice as high in jumping events compared to running events. For all of the analyzed disciplines, overall improvement rates were >50% higher for males than for females. The performance sex difference evolves from < 5% to 10-18% in all the analyzed disciplines from age 11 to 18 yr.

**Conclusion:**

To the authors’ knowledge, this is the first study to present absolute and relative annual performance developments in running and jumping events for competitive athletes from early to late adolescence. These results allow coaches and athletes to set realistic goals and prescribe conditioning programs that take into account sex-specific differences in the rate of performance development at different stages of maturation.

## Introduction

Running and jumping are fundamental motor skills in most sport disciplines. Such skills are developed throughout life via growth, maturation and training [1]. Males have consistently performed better in sports reliant on sprinting, jumping and endurance capacity [2–6]. As women have gradually been given the opportunity to compete on equal terms with men, the sex difference in performance has progressively narrowed before stabilizing in the 1980s [2,4,6]. Recent studies of world-class athletes indicate that the sex difference is 10–12% for running events [2,4,6] and ~19% for jumping events [6]. To date, no studies have documented when these sex differences arise. An understanding of the expected sex differences is important to allow coaches and athletes to set realistic goals and prescribe optimal conditioning programs that take into account sex-specific differences in the rate of performance development at different stages of maturation.

Sexual dimorphism during puberty is highly relevant for understanding sex-specific performance developments in sports. The initiation of the growth spurt in well-nourished girls occurs at about 9–10 yrs of age. Age at peak height velocity (PHV) and peak weight velocity (PWV) in girls is 11–12 and 12–13 yrs, respectively, with an average 7–9 cm and 6–9 kg annual increase [7,8,9]. The growth spurt and PHV in girls occurs approximately 2 years earlier than for boys. However, the magnitude of the growth spurt is typically greater in boys, as they on average gain 8–10 cm and 9–10 kg annually at PHV and PWV, respectively. Girls experience an escalation in fat mass compared to boys. Fat free mass (FFM) (also termed lean muscle mass) is nearly identical in males and females up to the age of 12–13 yrs. FFM plateaus in females at 15–16 years of age, but continues increasing in males up to the age of 19–20 yrs [7,8,9]. On average, boys and girls increase their FFM by 7.2 and 3.5 kg·year^-1^, respectively, during the interval near peak height velocity. Corresponding estimates for changes in absolute fat mass are 0.7 and 1.4 kg·year^-1^, while estimates for relative fatness are -0.5% and +0.9%·year^-1^ in boys and girls, respectively [8].

Previous cross-sectional studies of primarily non-competitive adolescents reveal that sex differences in physical capacities (assessed as $\dot{V}$ O_2_ peak or isometric strength in the majority of cases) are negligible prior to the onset of puberty [9–15]. During the adolescent growth spurt, however, marked sex differences develop. This can primarily be explained by hormone-dependent changes in body composition [8,16] and increased red blood cell mass in boys [16,17]. Despite such sex-specific differences having a pronounced effect on the training process, the consequences these dissimilarities should have for goal setting, planning and implementation of training for boys and girls of different ages, remains poorly understood. No published studies to date have investigated expected annual performance developments in running and jumping disciplines among competitive athletes, from the start through to the end of puberty.

Information regarding the realistic potential for development for a dedicated athlete from the start to the end of puberty is currently lacking in the research literature. Only longitudinal or mixed longitudinal data can provide adequate information regarding performance development during the adolescence growth spurt [14]. The Statistics Committee of the Norwegian Athletics Federation has, over several decades, recorded and systemized competition results from athletic events for all Norwegian athletes from the age of 11 years. This unique database provides the opportunity to investigate the long-term performance development of competitive athletes in sports which place high demands on endurance running, sprint running and jumping ability, from childhood up until the end of puberty. Hence, the purpose of this study was to quantify performance developments in athletic running and jumping disciplines in the age range 11–18 yr and identify possible progression differences as a function of age, discipline and sex.

## Materials and Methods

### Data sample

This study was conducted in accordance with the declaration of Helsinki. Since our data are based on publically available resources, no informed consent was obtained. This study was approved by the local ethics committee at the Norwegian University of Life Sciences.

The Norwegian Athletics Association annually publishes all-time best results categorized by sex, age and discipline [18]. Each record within these rankings documents performance, name, birth date, club, competition date and venue where the result was set. The database is restricted to the individual season best for each discipline. In the present study, the 100 all-time best male and female 60 m, 800 m, long jump and high jump athletes in each age category from 11 to 18 years were included for analysis. According to Norwegian track & field regulations, athletes are allowed to compete in these disciplines from the age of 11. Long distance disciplines were excluded from analyses as Norwegian athletes are not allowed to compete in such disciplines before the age of 15. Throwing and hurdling events were also excluded due to different competition regulation standards across age categories (i.e. weight of throwing implement, hurdle height, etc.). The included 100 all-time best lists contain data back to 1975. Only outdoor season data were included for 800 m, long jump and high jump, while 60 m included both indoor and outdoor data. However, 60 m results obtained without electronic timing were excluded. Only 60 m and long jump results obtained with legal wind speed (≤ 2 m·s^-1^) were included. Overall, the sample consisted of results from 1373 male and 1149 female athletes and a total of 6400 individual results. The distribution of male/female athletes across the analyzed sports disciplines were 408/369 for 60 m, 414/379 for 800 m, 440/371 for long jump and 354/347 for high jump. About 15–20% of the athletes were in the 100 all-time best lists in more than one discipline.

### Statistical analyses

The data were analyzed using mixed models with result as dependent variable and age as fixed explanatory variable with an athlete-specific random variable to account for within-athlete dependency. Separate analyses were performed by discipline and sex. We used estimated marginal means with 95% confidence limits (95% CIs) to produce plots of expected progression by gender and exercise. Descriptive statistics are presented as mean and standard deviation (SD) in addition to percent (%) for change. Effect magnitudes (based on Cohen’s *d*) across categories were interpreted categorically as small (*d* from 0.2 to 0.6), moderate (*d* from 0.6 to 1.2) or large (*d* from 1.2 to 2.0) using the scale presented by Hopkins et al. [19]. All analyses were performed using SAS software version 9.2 (SAS Institute Inc., Cary, NC, USA).

## Results

### 60 m sprint

**Fig 1:**
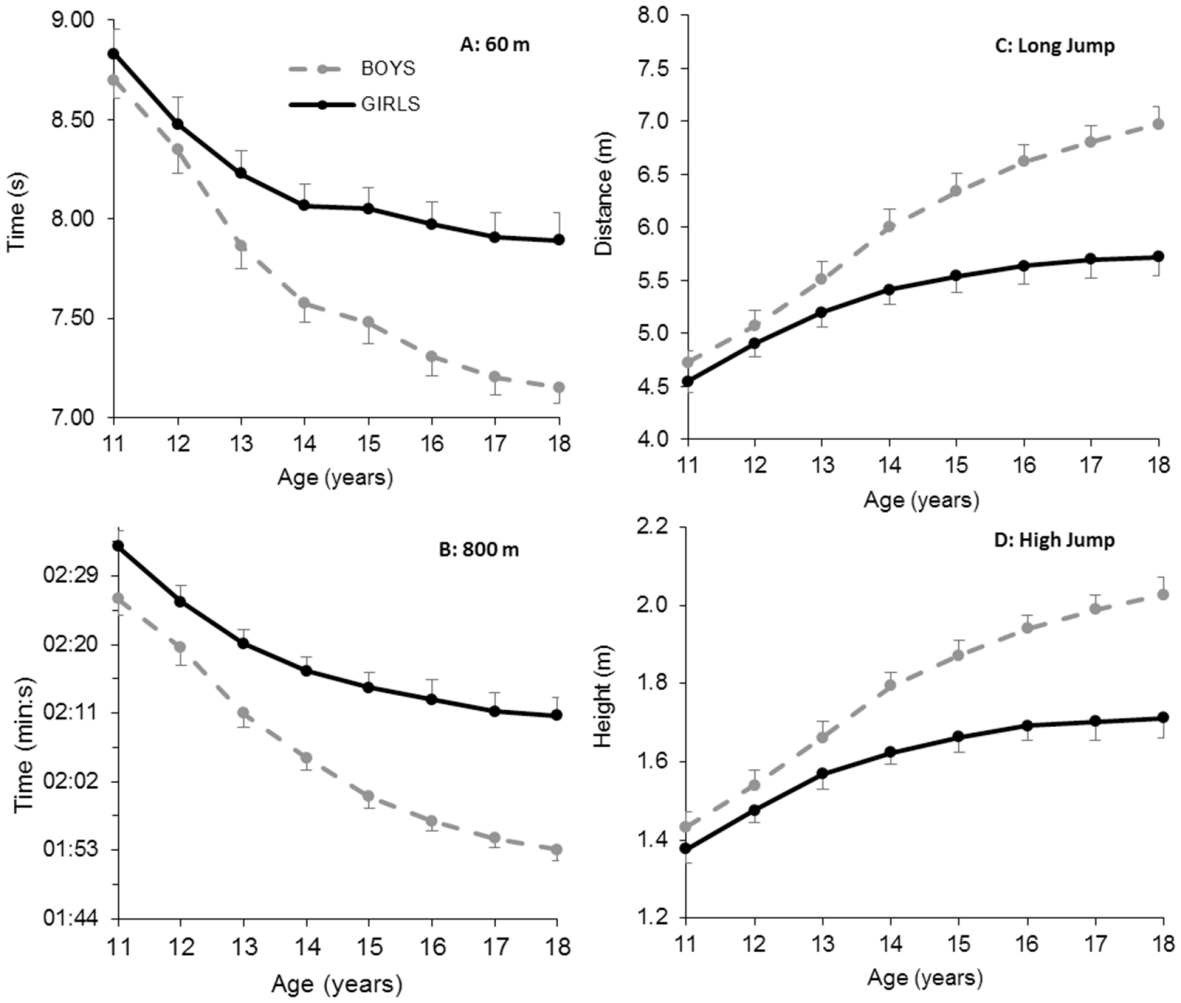
Performance development from age 11 to 18 in running and jumping disciplines. Data are mean ± SD for 60 m (panel A), 800 m (panel B) long jump (panel C) and high jump (panel D) for top 100 Norwegian male and female performers in each discipline.

**Fig 2:**
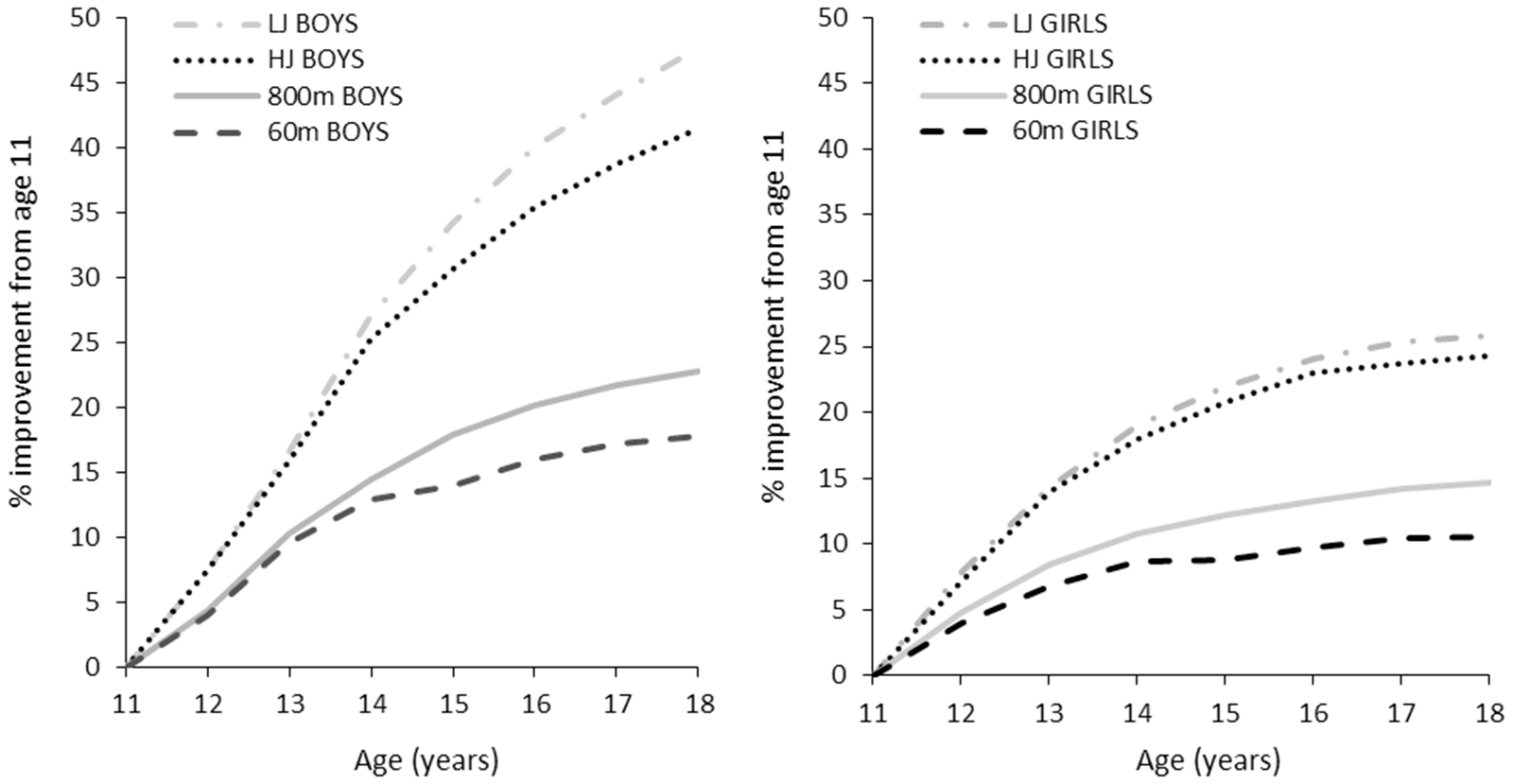
Percentage improvement in performance from age 11 to 18 in long jump, high jump, 60 m sprint and 800 m. Data are mean for top 100 Norwegian male (panel A) and female (panel B) performers in each discipline.

**Fig 3:**
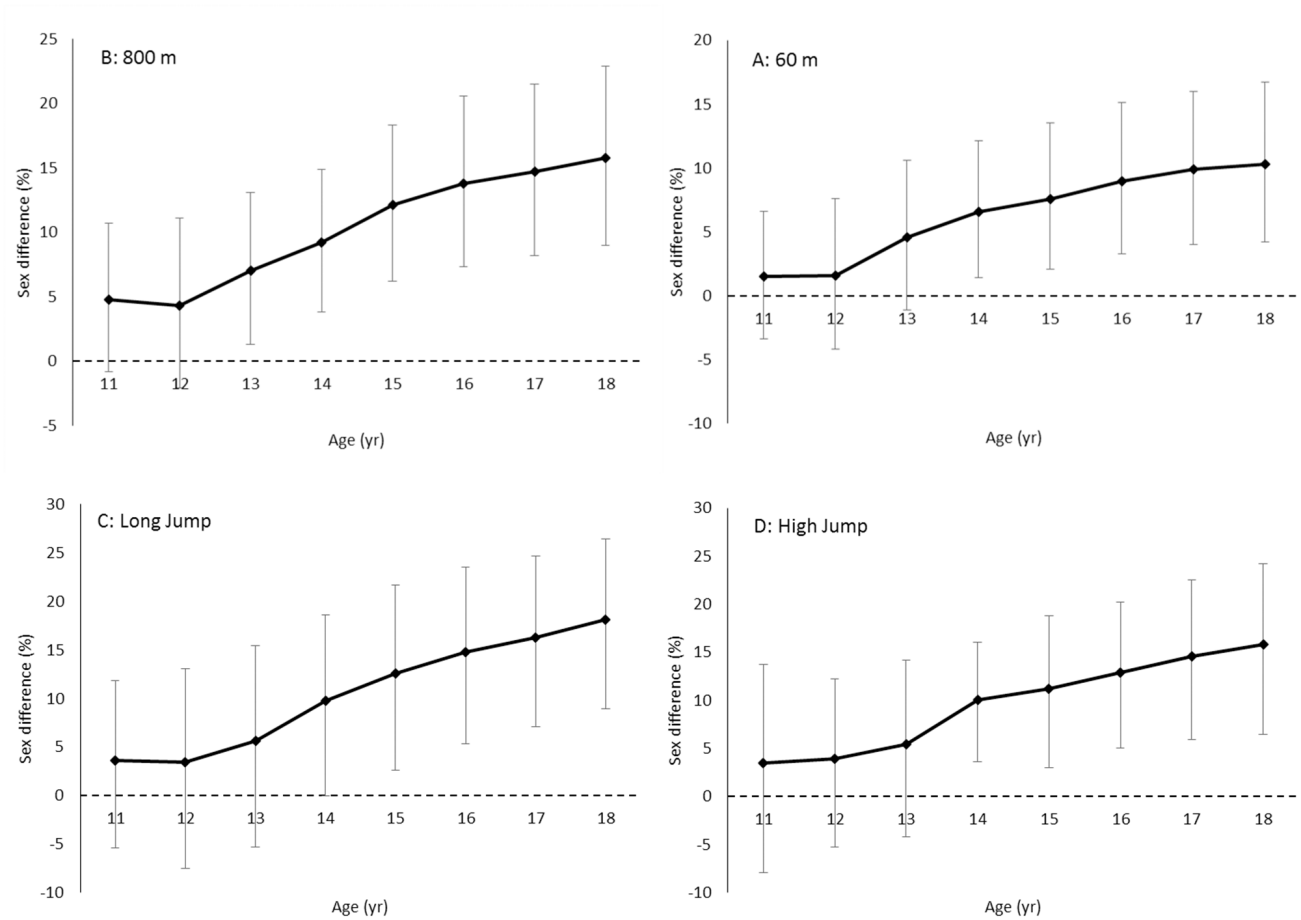
Sex difference (%) for performance in running and jumping disciplines from age 11 to 18. Data are mean and 95% CIs for 60 m (panel A), 800 m (panel B), long jump (panel C) and high jump (panel D) for top 100 Norwegian male and female performers in each discipline.

Table 1 and Fig 1 show that boys improve 0.3–0.5 s over 60 m sprint each year up to the age of 14 yr (very large to nearly perfect annual effect), 0.1–0.2 s annually from 14 to 17 yr (moderate to large annual effect), and 0.05 s from age 17 to 18 yr (moderate effect). Relative annual improvement peaks between 12 and 13 yr (5.8%; nearly perfect effect), and then gradually declines to 0.7% between age 17 and 18 yr (moderate effect). On average, boys improve their 60 m performance by 18% from age 11 to 18 yr (Fig 2). Girls improve 0.35 s over 60 m from age 11 to 12 yr (4%; very large effect) (Table 1 and Fig 1). Then, absolute and relative annual improvement gradually slows and almost plateaus between age 14 and 15 (0.02 s; 0.2%; trivial effect). From age 15 to 17, annual improvement increases somewhat to 0.07–0.08 s (~1%; moderate effect) before plateauing again between age 17 and 18 (0.02 s; 0.2%; trivial effect). In total, girls improve their 60-m performance by 11% from age 11 to 18 yr (Fig 2). Fig 3 (panel A) shows that the sex difference for 60 m sprint evolves from 1.5% at age 11 to 10.3% at the age of 18. Table 2 presents sex ratio in performance at each age stage, and shows that the sex ratio for 60 m running performance develops from 0.99 at age 11 to 0.91 at age 18.

**Table 1:** Expected progressions in running and jumping performance for 11–18 yr old males and females.

Age *(yr)*	60 m		800 m		Long Jump		High Jump	
	Boys	Girls	Boys	Girls	Boys	Girls	Boys	Girls
	*Progression*	*Progression*	*Progression*	*Progression*	*Progression*	*Progression*	*Progression*	*Progression*
	*(s and %)*	*(s and %)*	*(s and %)*	*(s and %)*	*m (%)*	*m (%)*	*m (%)*	*m (%)*
**11–12**	-0.35 (4.1)	-0.35 (4.0)	-6.4 (4.4)	-7.3 (4.8)	+0.35 (7.4)	+0.36 (7.9)	+0.11 (7.4)	+0.10 (7.2)
**12–13**	-0.48 (5.8)	-0.25 (2.9)	-8.7 (6.2)	-5.5 (3.8)	+0.43 (8.6)	+0.30 (6.0)	+0.12 (7.9)	+0.09 (6.3)
**13–14**	-0.29 (3.7)	-0.16 (2.0)	-5.9 (4.5)	-3.6 (2.6)	+0.50 (9.0)	+0.21 (4.1)	+0.13 (8.1)	+0.06 (3.6)
**14–15**	-0.10 (1.3)	-0.02 (0.2)	-5.2 (4.1)	-2.2 (1.6)	+0.34 (5.6)	+0.13 (2.4)	+0.08 (4.3)	+0.04 (2.4)
**15–16**	-0.17 (2.3)	-0.08 (1.0)	-3.2 (2.7)	-1.6 (1.2)	+0.28 (4.4)	+0.10 (1.8)	+0.07 (3.6)	+0.03 (1.8)
**16–17**	-0.10 (1.4)	-0.07 (0.8)	-2.3 (1.9)	-1.5 (1.2)	+0.19 (2.9)	+0.06 (1.1)	+0.05 (2.5)	+0.01 (0.6)
**17–18**	-0.05 (0.7)	-0.02 (0.2)	-1.5 (1.4)	-0.6 (0.4)	+0.17 (2.5)	+0.02 (0.4)	+0.04 (1.9)	+0.01 (0.5)

Data are mean (standard deviation) for top 100 Norwegian male and female performers in each discipline.

**Table 2:** Sex ratio in running and jumping performance for 11–18 yr old males and females.

	*60 m*	*800 m*	*Long Jump*	*High Jump*
**11**	0.99	0.95	0.96	0.97
**12**	0.98	0.96	0.97	0.96
**13**	0.96	0.93	0.94	0.95
**14**	0.94	0.92	0.90	0.90
**15**	0.93	0.89	0.87	0.89
**16**	0.92	0.88	0.85	0.87
**17**	0.91	0.87	0.84	0.85
**18**	0.91	0.86	0.82	0.84

Data are calculated from mean results of top 100 Norwegian male and female performers in each discipline.

### 800 m

Table 1 and Fig 1 show that boys improve 6–9 s over 800 m each year up to age 14 yr (very large to nearly perfect annual effect). Relative annual improvement peaks between age 12 and 13 (6.2%; nearly perfect effect), then gradually decreases to 1.5 s between age 17 and 18 (1.4%; moderate effect). On average, boys enhance their 800-m performance by 23% from age 11 to 18 (Fig 2). For girls, both absolute and relative annual performance development gradually decreases across the analysed age stages. The improvement is slightly above 7 s between age 11 and 12 yr (4.8%: very large effect), decreasing to only 0.6 s from age 17 to 18 (0.4%; small effect) (Table 1 and Fig 1). Fig 2 shows that girls enhance their 800-m performance by 15% from age 11 to 18. The 800 m performance sex difference evolves from 4.8% at the age of 11 to 15.7% at the age of 18 (Fig 3, panel B). Table 2 shows that the sex ratio for 800 m running performance develops from 0.95 at age 11 to 0.86 at age 18.

### Long jump

Table 1 and Fig 1 show that annual long jump improvement among boys gradually increases from 35 cm between age 11 and 12 yr (7.4%; very large effect) to 50 cm between age 13 and 14 (9%; very large effect). Both absolute and relative annual development then gradually falls to 17 cm between age 17 and 18 (2.5%; moderate effect). Fig 2 shows that boys, on average, improve their long jump performance by 48% from age 11 to 18 yr. For girls, both absolute and relative annual performance enhancement gradually falls from age 11 to 12 yr (36 cm; 7.9%; very large effect) until nearly plateauing between 17 and 18 yr (2 cm; 0.4%; trivial effect) (Table 1 and Fig 1). Overall, girls typically improve their long jump performance by 26% throughout the analysed age stages (Fig 2). The sex difference in long jump evolves from 3.6% at the age of 11 to 18% at the age of 18 (Fig 3, panel C). Table 2 shows that the sex ratio for long jump performance develops from 0.96 at age 11 to 0.82 at age 18.

### High jump

Table 1and Fig 1 show that boys improve their high jump performance by 11–13 cm each year up to the age of 14 (7–8%; very large annual effects). Both absolute and relative annual improvement peaks between age 13 and 14 (13 cm; 8.1%; very large effect), then gradually decreases to 4 cm from age 17 to 18 (1.9%; moderate annual effect). Overall, boys improve their high jump performance by, on average, 41% from age 11 to 18 (Fig 2). For girls, both absolute and relative annual improvement decreases from 10 cm from age 11 to 12 yr (7.2%; very large effect) until it plateaus from age 16 (1 cm; ~0.5%; small annual effects) (Table 1 and Fig 1). Overall, girls typically improve their high jump performance by 24% from age 11 to 18 (Fig 2). The sex difference in high jump performance evolves from 3.5% at the age of 11 to 16% at the age of 18 (Fig 3, panel D). Table 2 shows that the sex ratio for high jump performance develops from 0.97 at age 11 to 0.84 at age 18.

## Discussion

To the authors’ knowledge, this is the first study to present absolute and relative annual performance developments in running and jumping events for competitive athletes from early to late adolescence. Data from the 100 best performers in each age category from age 11 to 18 years show that male and female athletes perform almost equally up to the age of 12. Beyond this age, males outperform females. Relative annual performance development in females gradually decreases throughout the analyzed age period. In males, annual relative performance development accelerates up to the age of 13 (for running events) or 14 (for jumping events) and then gradually declines when approaching 18 years of age. The relative improvement from age 11 to 18 was twice as high in jumping events compared to running events. For all of the analyzed disciplines, overall improvement rates were >50% higher for males than for females.

Sexual dimorphism during puberty is central in understanding the sex- and discipline-specific performance developments observed in the current study. During puberty, boys begin to produce higher levels of circulating testosterone. This affects the production of muscle fibers through direct stimulation of protein synthesis [16,17]. Higher testosterone levels result in more muscle mass, which in turn facilitates greater power production and more advantageous ground reaction forces during running and jumping. Adolescent weight gain in boys is principally due to increased height (skeletal tissue) and muscle mass, while fat mass remains relatively stable [7,8,9]. In contrast, during puberty girls begin to produce higher levels of circulating estrogen and other female sex hormones [16]. Compared to their male counterparts, they experience a less pronounced growth spurt and a smaller increase in muscle mass, but a continuous increase in fat mass, thereby lowering the critical ratio between muscular power and total body mass. Fat free mass (FFM) in males and females is nearly identical up to 12–13 years of age. Subsequently, FFM plateaus in females at 15–16 years of age, but continues to increase in males up to 19–20 years of age [7,8,9]. On average, boys and girls increase their FFM by 7.2 and 3.5 kg·year^-1^, respectively, during the interval near peak height velocity. Corresponding estimates for changes in absolute fat mass are 0.7 and 1.4 kg·year^-1^, while estimates for body fat percentage are -0.5% and +0.9%·year^-1^ in boys and girls, respectively [8]. Moreover, increased red blood cell mass in boys at this time may contribute towards the widening sex difference for 800 m running [16,17]. Taking present and previous findings together, there appears to be a strong mechanistic connection between the observed sex-specific performance developments and hormone-dependent changes in body composition during puberty. Figs 1 and 2 are very consistent with the muscle mass proportion curve outlined by Malina et al. [8], where a sex difference break point at the age of 12 is clearly present. Beyond this age, males outperform females because maturation results in a shift in body composition. Our results are in line with previous investigations exploring physical capacities such as $\dot{V}$ O_2 peak_ and isometric strength in non-competitive or non-specialized adolescents [8–12,20].

Fig 3 shows that performance development during puberty varies considerably according to sport discipline. Within the jumping disciplines, improvement rates were slightly higher for long jump compared to high jump. Within the running disciplines, the improvement rates were slightly higher for 800 m compared to 60 m. Magnitudes of improvement within sex in jumping events were twice as large as in running events throughout the analyzed age stages. Moreover, tables 1 and 2 show that the peak rate of improvement in boys is reached at a later stage in jumping events (13–14 years) compared to running events (12–13 years). From a motor learning perspective, running can be thought of as largely inherited, while jumping skills are to a larger degree affected by practice [21]. The relatively low performance progress in running exercises can possibly be explained, at least in part, by the fact that running is an innate movement typically developed naturally through growth and maturation from 1–2 years of age. A large part of the performance potential has thus already been exploited through natural play, growth and maturation. Jumping is also an innate movement, but not in the way in which long jump and high jump are executed in track and field athletics. These are performed with an approach speed from 7 to 12 m·s^-1^, and with the athlete required to handle forces of up 10 times their body weight [22,23]. It can therefore require many years of specific training to learn an optimal approach, take-off, flight- and landing technique. The relatively greater progress in jumping exercises can also be explained by growth and increased body height during puberty. The increase in body height means that the center of gravity will be higher, providing better mechanical conditions for performance in jumping events. Furthermore, Beunen & Malina [1] suggest that the plateau for some motor tasks occurs at a slightly older age. Previous studies of non-competitive adolescent boys indicate that peak gains in strength and power typically correspond with peak gains in body mass and muscle mass. Conversely, peak gains for running velocity and aerobic power typically occur at peak height velocity or slightly before [1,11,15] The adolescent performance spurt for running speed is likely related to growth of the lower extremities, as the legs experience maximum growth before trunk, neck and head [24].

The current results indicate that the sex difference evolves from < 5% to 10–18% in all the analyzed disciplines from age 11 to 18 yr. The gap widens considerably during early adolescence before gradually stabilizing when approaching the age of 18. This evolution is practically identical for the running and jumping disciplines. The observed sex differences at the age of 18 are in line with previous studies of world-class athletes where a sex difference of 10–12% for running events [2,4,6] and ~19% for jumping events [6] has been reported. To the authors’ knowledge, this is the first study to document when these sex differences arise.

A limitation of the present study is the lack of maturity status data. Youths who are successful in sport may differ in maturity status compared with the general population. Even at a later age, early maturing boys continue to have an advantage in strength and power tasks, while the later maturing boys typically catch-up when it comes to speed tasks [25]. In girls, maturity-associated variation in performance is not consistent among tasks and from age to age [9,26]. Most samples of female adolescent athletes have mean or median ages at menarche within the normal range, with the exception of gymnasts, ballet dancers, figure skaters and divers, who tend to be later than those of athletes in other sports [1]. In Belgian male track athletes, all 15–16 year old athletes except one had a skeletal age in advance of chronologic age, while in corresponding 17–18 years old athletes, two-thirds had skeletal age equal to or in advance of that expected for chronologic age [27]. However, it is worth noting that regular training does not affect growth, the timing and magnitude of peak height velocity, and skeletal and sexual maturation in young athletes [28] and the pubertal progress of boys and girls active in sport is similar to the progress observed in boys or girls not active in sport.

Another limitation is that only a handful of the present athletes were top 100 performers in every age category from 11 to 18 years of age. One could argue that early maturing athletes are overrepresented in the younger age stages, while they are successively replaced with average or late maturing athletes who catch up or outperform the early maturers when approaching late adolescence. Thus, the present data might overestimate individual annual performance development. However, falling trends among early maturers, at least up until a point where they are no longer be within the top 100, are accounted for by the mixed models analysis. Furthermore, analysis of the small sample of athletes who were top 100 performers in every age category (2–6 athletes in each discipline) reveal similar performance development as our mixed model analysis. When interpreting the current data, it should also be recognized that there are some limitations associated with using ratios and percentage change values [29]. Since the slope of the relationship between the logarithmically transformed numerator and denominator deviated somewhat from one for some variables, these ratios may not scale accurately towards the extremes of each range. However, mixed model analyses were performed on absolute rather than ratio values. Furthermore, percentage change and ratio data are presented supplemental to absolute values, and should therefore facilitate rather than mislead interpretation.

Because of the physical changes that occur during puberty, the optimal training program for this age group likely differs somewhat between boys and girls. For example, for adolescent girls, increases in fat mass and reductions in relative strength often occur alongside reductions in coordination and neuromuscular control [30]. This may impair training tolerance and increase the risk of certain types of injury [31]. Hence it may be beneficial for female athletes to have a greater focus on neuromuscular training during this period [32]. From a practical perspective, the present results allow coaches and athletes to set realistic goals and prescribe conditioning programs that take into account sex-specific differences in the rate of performance development at different stages of maturation. Because hormone-dependent changes during puberty mean that adolescent boys and girls respond somewhat differently to exercise, it is important that training is tailored based on sex and the specific stage of growth and maturation.
